# Protocol: Streamline cloning of genes into binary vectors in *Agrobacterium *via the Gateway^® ^TOPO vector system

**DOI:** 10.1186/1746-4811-4-4

**Published:** 2008-01-22

**Authors:** Ruqiang Xu, Qingshun Quinn Li

**Affiliations:** 1 Department of Botany, Miami University, Oxford, OH 45056, USA; 2 *Present address:* Department of Anatomy and Neurobiology, Virginia Commonwealth University, Richmond, VA 23298, USA

## Abstract

**Background:**

In plant functional genomic studies, gene cloning into binary vectors for plant transformation is a routine procedure. Traditionally, gene cloning has relied on restriction enzyme digestion and ligation. In recent years, however, Gateway^® ^cloning technology (Invitrogen Co.) has developed a fast and reliable alternative cloning methodology which uses a phage recombination strategy. While many Gateway- compatible vectors are available, we frequently encounter problems in which antibiotic resistance genes for bacterial selection are the same between recombinant vectors. Under these conditions, it is difficult, if not sometimes impossible, to use antibiotic resistance in selecting the desired transformants. We have, therefore, developed a practical procedure to solve this problem.

**Results:**

An integrated protocol for cloning genes of interest from PCR to *Agrobacterium *transformants via the Gateway^® ^System was developed. The protocol takes advantage of unique characteristics of the replication origins of plasmids used and eliminates the necessity for restriction enzyme digestion in plasmid selections.

**Conclusion:**

The protocol presented here is a streamlined procedure for fast and reliable cloning of genes of interest from PCR to *Agrobacterium *via the Gateway^® ^System. This protocol overcomes a key problem in which two recombinant vectors carry the same antibiotic selection marker. In addition, the protocol could be adapted for high-throughput applications.

## Introduction

With the rapid progress of many genome sequencing projects in plants, plant scientists are stepping up the pace of gene function studies. To this end, both gene cloning and subcloning have become routine procedures that traditionally rely on restriction enzyme digestion and ligation. In recent years, however, Gateway^® ^cloning technology (Invitrogen Co.) has developed a fast and reliable alternative cloning methodology based on bacteriophage λ site-specific recombination. In plant functional genomics research, the gene of interest usually needs to be cloned into binary vectors of a larger size (5 to 12 kb) in order to obtain transgenic plants via *Agrobacterium*-mediated T-DNA transformation. Thus, in terms of its efficiency when compared to traditional DNA cloning, Gateway^® ^cloning technology has proved to be extremely useful for gene cloning into a larger size of vectors [1,2]. Many Gateway^® ^compatible binary vectors have been made available [1,2], including the pMDC series of binary vectors, which is freely available for noncommercial use. These vectors can be used for functional analysis of genes by constitutive or inducible ectopic expressions, antisense or RNAi expressions, promoter analyses, subcellular localizations, or complementation analyses [1].

In general, the Gateway^® ^technology involves a two-step process [3]. The gene of interest is first cloned into an Entry vector through the so-called BP reaction, which produces an Entry clone. When making the Entry clone, it is necessary to change the sequences of the gene's ends in order to be Gateway-compatible (recombinase recognition sites), but no restriction enzyme is involved during the entire cloning process. The system also takes advantage of the negative selection marker *ccd*B gene to eliminate the original vector after transformation [4]. Next, the resulting cloned gene is subcloned into one of the Destination Vectors by the LR reaction between the Entry clone and the Destination vector. Thus, once an Entry clone with the gene of interest has been made, a further advantage of the Gateway technology lies in the ease of subcloning it into a wide variety of Destination vectors through the LR reaction.

When using these vectors in conjunction with the Gateway^® ^cloning technology, however, we frequently encounter a situation in which antibiotic resistance genes for bacterial selection are the same between two recombinant vectors. Under these conditions, it is impossible to use the antibiotic resistance for the purpose of selecting desired transformants during cloning processes. One recently reported solution uses a restriction enzyme to digest the recombinant by-product [5], but it may often be impractical to select a unique restriction enzyme, especially when dealing with a large plant gene insert. Here, we present a method that uses differential selections of plasmids based on their replication origins and that, consequently, bypasses the problem indicated above.

We are particularly interested in expressing various plant genes encoding mRNA polyadenylation factor subunits through *Agrobacterium*-mediated transformation for gene functional studies. In our previous work [6], we created Entry clones by first generating a PCR product containing *att*B sites, which required two overlapping PCR reactions due to the addition of a 25 bp *att*B site on both ends of the PCR product. This *att*B-PCR product was then used in BP reactions with a donor vector pDONR™201 to generate the Entry clone. Since the pENTR™/D-TOPO^® ^cloning kit (Invitrogen) has now become available, only a 4-bp (CACC) leader needs to be added to the 5'-PCR primer for gene amplification. This allows the PCR product to be directionally cloned into the TOPO vector to generate an Entry clone. Thus, the TOPO technology allows us to easily produce Entry clones.

However, a major problem still remains and involves the bacterial selection marker for the TOPO vector and the binary destination vector pMDC series [1]. Since these vectors all harbor a kanamycin antibiotic-resistant gene for transformant selections, the antibiotic selection will not work when screening the recombinant clones after the LR reaction. One convenient solution would be to change a TOPO vector, but, based on our consultations with Invitrogen, there is no other directional TOPO Entry clone vector available. Alternatively, as noted above, the use of a restriction enzyme to digest the undesired plasmid after the LR reaction has been reported [5]. However, this solution is flawed because it involves the selection of an enzyme that only digests the recombinant by-product plasmid, not the target gene and the binary vector. This may be impractical, in some cases, especially with large plant genes. Therefore, we report here our development of a streamlined procedure which is based on the characteristics of the Gateway vectors, but which avoids all of the potential problems described above.

## Materials

### Reagents and Consumables

Template DNA or cDNA for PCR amplification of the gene of interest.

PCR Primers – the 5' end of the forward primer should add the 4 nucleotides, CACC, which are required for directional cloning using the pENTR/D-TOPO^® ^cloning kit.

High fidelity DNA polymerase (e.g., *Pfu *DNA polymerase) and appropriate 10× PCR buffer.

dNTPs

The PCR product and gel purification kit (e.g., QIAquick^® ^from Qiagen Inc.).

The pENTR/D-TOPO^® ^Cloning kit (Invitrogen Cat#KNM4500-01).

Chemically competent (or electro-competent, optional) *E. coli *strain, any *rec*A, *end*A *E. coli *strain, e.g., One Shot^® ^TOP10, DH5α™, XL-1 Blue or equivalent.

Gateway-compatible binary vectors (e.g., the pMDC series), which are freely available for noncommercial use at the Arabidopsis Biological Resource Center [7].

Dry ice

Ethanol

Lysozyme

CaCl_2_

LB liquid medium (10 g/L bacto-trypton, 5 g/L yeast extract, 10 g/L NaCl) and plates (LB + 15 g/L agar) supplemented with antibiotics (kanamycin, 50 μg/mL), as appropriate.

YEP liquid medium (10 g/L bacto-trypton, 10 g/L yeast extract, 5 g/L NaCl) and plates (YEP + 15 g/L agar) supplemented with antibiotics (kanamycin, 50 μg/m L), as appropriate.

Plasmid miniprep kit (e.g., Bio-Rad Quantum Prep^®^)

The Gateway^® ^LR Clonase™ enzyme mix kit (Invitrogen)

TE buffer, pH8.0

1.5 ml centrifuge tubes

Sterile culture tubes

### Equipment

PCR thermal cycler

Long wavelength UV transilluminator – long wave ultraviolet light is less damaging to DNA during excision of bands from gels.

42°C water bath (or Electroporator, e.g., Bio-Rad E. coli Pulser, if using electro-competent *E. coli *strains).

Horizontal gel electrophoresis equipment

Microcentrifuge

Shaking and non-shaking temperature-controlled incubators

## Protocol

**Figure 1:**
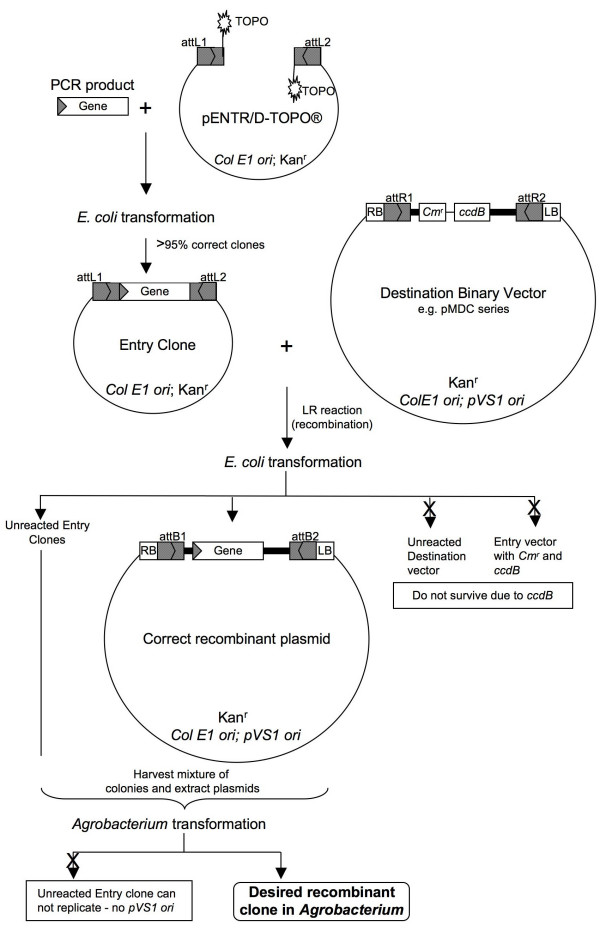
Schematic from PCR gene cloning to *Agrobacterium *expression clones using the Gateway^® ^cloning technology. *Col E1 ori*: origin of replication for *E. coli*. pVS1 ori: origin of replication for *Agrobacteria*. Kan^r^: kanamycin resistance gene. Cm^r^: chloramphenicol resistance gene. RB: T-DNA right border. LB: T-DNA left border. *att*L, *att*R, *att*B: recombination sites. *ccdB: E. coli ccdB *gene (note: the ccdB protein interferes with *E. coli *DNA gyrase and inhibits growth of most *E. coli *strains, e.g., DH5α™).

The following protocol was developed based on two sets of facts. First, the Gateway-compatible pMDC vectors contain the *ccd*B gene (as do other Destination vectors) whose protein product interferes with *E. coli *DNA gyrase [4], thereby inhibiting the growth of most *E. coli *strains used in the lab, such as DH5α™, TOP10 or XL-1 Blue. When recombination occurs between an Entry clone and a Destination vector, the *ccd*B gene on the Destination vector is replaced by the gene of interest. Significantly, cells that take up non-reacted vectors carrying the *ccd*B gene, or the by-product molecules retaining the *ccd*B gene, will not grow after transformation to these *E. coli *strains. Second, each of the pMDC series binary vectors harbors both *Col E1 *and pVS1 replication origins for *E. coli *and *Agrobacterium*, respectively [2]. In contrast, the pENTR vector can only replicate in *E. coli*, not in *Agrobacterium*, which is critical for selecting the binary vectors in *Agrobacterium *when transformed with a mixture of pENTR and the binary vectors. Thus, even if both vectors carry the same kanamycin selection, using the unique feature noted above will enable their differentiation after transforming to *Agrobacterium *in which the pENTR vector fails to replicate. Hence, by combining the commercial availability of cloning products with these vector characteristics, we have devised an efficient and novel pipeline which carries forward the entire process from cloning a gene of interest to producing an *Agrobacterium *transformant. As such, this same concept can be applied to solve similar problems in other vector systems or be scaled up for high-throughput processing. The schematic diagram of this protocol is presented in Figure 1.

### A. Creating the Entry clone using pENTR™ Directional TOPO^® ^cloning kit

Using pENTR/D-TOPO^® ^cloning kit, the genes, or DNA fragments of interest, are PCR- amplified and readily introduced into pENTR™ D-TOPO^® ^cloning vector to generate the Entry clone. The key steps are as follows:

1. Design PCR primers for gene amplification, with an addition of 4-nucleotide CACC at the 5' end of the forward primer.

2. Perform PCR to amplify the gene of interest with high fidelity DNA polymerase.

3. Run agarose gel electrophoresis to check PCR product, after which the desired band is cut and purified using the QIAquick^® ^Gel Purification Kit (Qiagen) or other substitutes.

4. Use the pENTR™ Directional TOPO^® ^cloning kit to set up TOPO^® ^cloning reaction: mix the PCR product with the pENTR™ TOPO^® ^vector (0.5:1 to 2:1 molar ratio) in the reaction buffer and incubate 5 minutes at room temperature, according to manufacturer's recommendation.

5. Take 2 μL of the TOPO^® ^cloning reaction mixture to transform competent *E. coli *cells (e.g., TOP10, DH5α™, XL-1 Blue), using heat shock at 42°C, spread the cells on LB plate containing 50 μg/mL kanamycin, and incubate overnight at 37°C.

6. Select 5–10 colonies and culture them by shaking overnight at 37°C in 2 mL LB liquid media containing 50 μg/mL kanamycin.

7. Isolate plasmid DNA by the plasmid miniprep kit.

8. Confirm the insert in the plasmids by restriction analysis or PCR or DNA sequencing. This is the Entry clone.

### B. Performing the LR recombination reaction

The Gateway^® ^LR Clonase™ enzyme mix kit (Invitrogen) was used to perform the LR recombination reaction:

1. Add the following components to a 1.5 mL microcentrifuge tube and mix:

Entry clone (100–300 ng) 1–5 μL

Destination vector, e.g., pMDC (150 ng/μL) 1 μL

5× LR Clonase Reaction Buffer 2 μL

TE buffer, pH8.0 to 8 μL

2. Add 2 μL LR Clonase™ enzyme, mix well by votexing briefly twice and spin down. Then, incubate the reaction mixture at 25°C for 1 hour to overnight.

3. To stop the LR reaction, add 1 μL of the Proteinase K (2 μg/μL) solution and incubate for 10 minutes at 37°C.

4. Take 3–5 μl of the LR reaction mixture to transform competent *E. coli *cells (e.g., TOP10, DH5α™, XL-1 Blue), spread the transformation on LB plate containing 50 μg/ml kanamycin and incubate overnight at 37°C. **Note**: *do not transform the Gateway^® ^LR reaction mixture into E. coli strains that contain the F2 episome (e.g., TOP10F2). These strains contain the ccdA gene and will prevent negative selection with the ccdB gene. Alternatively, transformants can also be selected by liquid culture with the antibiotic without spreading on a plate, and the selected culture can be directly subject to plasmid isolation*.

5. Harvest all colonies from the plate and isolate plasmid DNA mixture containing the non-reacted plasmids of the Entry clone, as well as the recombination plasmid between the Entry clone and the Destination vector.**Note**: *E. coli cells containing non-reacted plasmids of the Destination vector and by-product plasmids of the LR recombination reaction fail to grow because these plasmids contain the ccdB gene which is toxic to the cell growth of E. coli stain, e.g., TOP10, DH5α™, XL-1Blue *[3].

### C. Performing Agrobacterium transformation to select the recombinant expression clone

The choice of *Agrobacterium *transformation methods, e.g., chemical transformation vs. electroporation, is solely at the discretion of the lab. In our lab, chemically competent cells were used for transformation by the freeze-thaw procedure as described below.

1. Preparation of chemically competent *Agrobacterium *cells.

- Grow a colony of *Agrobacterium *in 2 mL YEP liquid media by incubating at 28°C overnight with shaking (~250 rpm).

- Transfer the cells to 50 mL YEP liquid media and incubate for 3 ~ 4 hr with shaking until OD_600 _= 0.5 ~1.0.

- Chill the culture on ice for 5 min, then centrifuge at 3000 × g for 5 min at 4°C and discard the supernatant.

- Rinse the cell pellet with 10 mL of 20 mM ice-cold CaCl_2 _and spin briefly again.

- Add 1 mL of 20 mM ice-cold CaCl_2 _to resuspend the cells gently on ice.

- Take an aliquot of 0.1 mL of the competent cells into a pre-chilled microcentrifuge tube.

**Note**: *Freeze any extra aliquots in liquid nitrogen or dry ice-EtOH slurry and store at -70°C for future use*.

2. Add 1 μg or more plasmid DNA mixture (Step 2.4) to the 0.1 mL of competent cells, mix gently, but thoroughly, and then freeze in liquid nitrogen or dry ice-EtOH slurry. **Note**: *When using -70°C stored competent cells, thaw the cells until just becoming liquid before adding plasmid DNA*.

3. Thaw the cells in a 37°C water bath for 5 min. Add ~150 μL YEP liquid media and incubate at 28°C for 2~4 hr with gentle shaking; then transfer and spread all cells on YEP plate containing 50 μg/mL kanamycin and/or other antibiotics, if appropriate, and incubate 2 to 3 days at 28°C. **Note**: *Since the unreacted Entry clones are removed by the selection due to lack of Agrobacterium replication origin, only the recombinant Destination plasmid can be selected in Agrobacterium*.

4. The positive colonies will grow. **Note**: *According to our experience, all the positive colonies contain the desired recombination plasmid*.

5. [Optional] verification of the positive colonies can be done by a colony PCR method. If desired, plasmid can be isolated and used for verification by restriction analysis or DNA sequencing. **Note**: *For isolation of the binary plasmid from Agrobacterium, culture a single colony in YEP liquid media overnight at 28°C. Then, we use Quantum Prep^® ^Plasmid Miniprep kit (Bio-Rad) to isolate plasmid DNA with the following minor modifications: prior to cell lysis, add 10 μL freshly-prepared solution of lysozyme (100 mg/mL in 10 mM Tris-HCl, pH8.0) into the cell suspension followed by incubation for 5 minutes at room temperature. As the final step, elute plasmid DNA with pre-heated TE buffer at 70°C. In case a low yield or low quality plasmid DNA is obtained from Agrobacteria, the plasmid can be transformed into E. coli to produce sufficiently pure plasmid DNA for sequencing purposes*.

6. The *Agrobacterium *colonies are ready to be used for plant transformation.

### Examples of successful application

**Figure 2:**
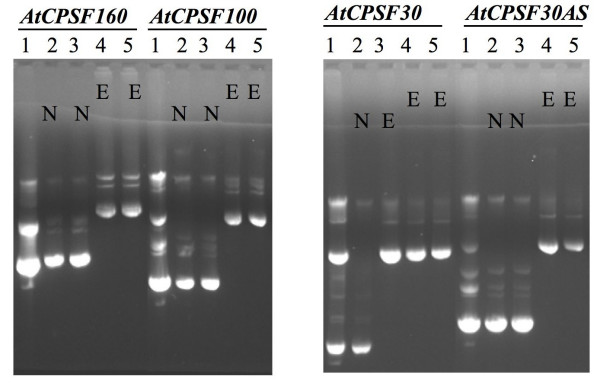
Patterns and identities of plasmids isolated from *E. coli *and *Agrobacterium *transformants, respectively. Plasmid DNAs were separated on 0.6% agarose gel with ethidium bromide staining. *AtCPSF160, AtCPSF100, AtCPSF30 *and *AtCPSF30AS *are four genes used for the cloning work in this report. Lane 1: plasmid DNAs isolated from a pool of the LR reaction mixture-derived *E. coli *transformants. Lanes 2 and 3: plasmid DNAs isolated from each randomly selected single colony of the LR reaction mixture-derived *E. coli *transformants. Lanes 4 & 5: plasmid DNAs isolated from each randomly selected single colony of *Agrobacterium *transformants. N: plasmid identity of corresponding lane was Entry clone confirmed by DNA sequencing. E: plasmid identity of corresponding lane was Expression clone confirmed by DNA sequencing.

Using this streamlined method, we were initially successful in cloning four polyadenylation factor genes [6]. Subsequently, other lab members have successfully followed the same method to clone many different genes (D. Xing, H. Zhao, X. Ye, and Q.Q. Li, unpublished results), and it is now routinely used in our laboratory. Figure 2 shows the patterns and identities of plasmids and/or clones derived from different cloning steps of the method during the cloning of four polyadenylation factor genes (*AtCPSF160, AtCPSF100, AtCPSF30 *and *AtCPSF30AS*). The plasmid DNAs isolated from a pool of *E. coli *transformants by the mixture of LR reaction between the Entry clone and the Destination vector contained two types of plasmids: the unreacted Entry clones and the desired expression clones from the LR reaction (Figure 2, lane #1 in each panel). When two individual clones were randomly selected from these mixtures of transformants for plasmid sequencing, only one out of eight clones from the four genes was found to be the desired expression clone, and all others were the unreacted entry clones (Figure 2, lanes # 2 & 3). In contrast, when plasmid DNAs from a pool of transformants were generated in the competent *Agrobacterium *cells by the same mixture (of LR reaction between the Entry clone and the Destination vector), all the resulting randomly chosen transformants were the desired expression clones (Figure 2, lanes # 4 & 5). We presumed this was because the unreacted Entry clones failed to propagate in the *Agrobacterium *cells due to the difference of replication origin of the Entry clones. These *Agrobacteria *were ready for plant transformation.

## Comments

When two recombination vectors carry the same antibiotic resistance gene, it is difficult, if not sometimes impossible, to use antibiotic resistance in selecting the desired transformants. Our protocol solves this problem by employing the Gateway^® ^cloning technique to produce expression clones for *Agrobacterium*-mediated plant transformation. Moreover, our protocol improves upon previous methods by utilizing a more straightforward strategy which eliminates the potentially time-consuming step of selecting an appropriate enzyme to digest and remove the unreacted Entry clones from the LR reaction, as previously suggested [5]. Our experience indicates that this is an efficient and reliable method for cloning genes of interest by PCR and introduced into *Agrobacterium *for plant functional genomic studies. Finally, our protocol offers the potential for use in high-throughput applications because many of the reactions can be modified for parallel processing.

## Competing interests

The author(s) declare that they have no competing interests.

## Authors' contributions

RX conceived, designed and carried out the experiments reported in this study. RX drafted the manuscript and QQL analyzed the results and finished the manuscript. Both authors read and approved this final version of the manuscript.
